# Research progress in modulating the auditory system by the cochlear circadian clock system in response to noise exposure

**DOI:** 10.3389/fnins.2025.1507363

**Published:** 2025-03-18

**Authors:** Xiaoqiong Song, Mengzhu Cheng, Cui Gu, Fenghan Wang, Kefeng Ma, Chunping Wang, Xiaojun She, Bo Cui

**Affiliations:** ^1^School of Public Health, Shandong Second Medical University, Weifang, Shandong, China; ^2^Academy of Military Medical Sciences, Tianjin, China; ^3^School of Public Health and Management, Binzhou Medical University, Yantai, Shandong, China; ^4^School of Chinese Medicine, Tianjin University of Traditional Chinese Medicine, Tianjin, China

**Keywords:** cochlea, circadian clock, circadian rhythm, noise, noise-induced hearing loss

## Abstract

The circadian clock is an endogenous system evolved to adapt to environmental changes. Recent studies confirmed that the cochlea exhibits circadian oscillations regulating auditory function. These oscillations are linked to brain-derived neurotrophic factor and glucocorticoid levels. Circadian rhythms influence cochlear sensitivity to noise by regulating the secretion of brain-derived neurotrophic factors and glucocorticoids. This study explores the regulatory mechanism of the circadian clock system, its impact on the auditory system, and its potential role in noise-induced hearing loss. Understanding the regulatory mechanisms of circadian rhythms in auditory function will provide new ideas for developing treatments for noise-induced hearing loss.

## 1 Introduction

The evolution of life is closely tied to the Earth’s rotation, with many organisms developing internal timing mechanisms known as circadian clocks to respond to environmental changes. To adapt to the cyclic environmental variations, an organism’s biological processes, including body temperature, hormone levels, sleep, and wakefulness, exhibit a regular circadian cycle of approximately 24 h, which is termed the circadian rhythm. Circadian rhythms regulate the timing of bodily functions, and are essential for maintaining organ and tissue homeostasis. In mammals, the circadian clock is divided into the central and peripheral clocks (Schibler, 2005). The central clock, located in the suprachiasmatic nucleus (SCN) of the hypothalamus, acts as the “central pacemaker” controlling circadian rhythms (Brown and Piggins, 2007). Almost every tissue and organ contain a peripheral clock, which are coordinated by the central clock to jointly maintain the circadian rhythm of organisms (Zhang et al., 2014). For instance, circadian rhythms in cardiovascular cells regulate a variety of cardiovascular functions such as endothelial function, thrombosis, and heart rate (Crnko et al., 2019); circadian rhythms in hepatocytes are involved in regulating liver metabolism to maintain homeostasis (Mukherji et al., 2019); and in the kidneys, renal plasma flow, glomerular filtration rate, and tubular reabsorption and/or secretory processes are all related to the renal circadian clock (Firsov and Bonny, 2018).

The cochlea, as a peripheral auditory organ, also has a circadian clock system (Loudon, 2014). Further research is therefore required to study the cochlear circadian clock system and its regulatory mechanisms on the auditory system. Additionally, sound, as an organism-perceivable environmental factor, may act as a *zeitgeber* to regulate the cochlear circadian clock system, influencing auditory functions and even pathophysiological processes. However, there is a lack of clarity about how the cochlear biological clock system is involved in and regulates the processes of NIHL. In this review, we discuss the role of circadian rhythms in the auditory system and provide an overview of the latest advances in the understanding of circadian cochlear circadian clock system. Furthermore, we describe how circadian rhythms are related to noise-induced hearing loss (NIHL).

## 2 The circadian clock system

The circadian clock system exists in every cell of the mammalian organism, regulating behavior and physiological processes to maintain an internal sense of time without any external cues. The circadian clock system regulates a tightly coupled circadian rhythm that governs the sleep-wake cycle, metabolism, feeding, immune response, blood pressure, hormone secretion, and other bodily functions (Gachon et al., 2004).

### 2.1 The central and peripheral circadian clock

The central circadian clock synchronizes the circadian clock in the body with light and dark conditions in the external environment by regulating the circadian clock genes expression (Webb et al., 2009). The peripheral circadian clock, located in several organs and tissues including the liver, spleen, lungs, and heart, generates self-sustaining oscillations in tissues in a precise pattern, prompting different biological pathways to operate at specific times of the day to enhance the body’s functional responses (Buijs and Kalsbeek, 2001; Basinou et al., 2017; Fontana et al., 2019).

There is a strong association between the central and peripheral circadian clock. The SCN interacts with the circadian clocks in the peripheral organs and tissues via the autonomic nervous system or humoral signals such as glucocorticoids (GCs) (cortisol in human and corticosterone in rodent). McDearmon et al. (2006) and Husse et al. (2011) demonstrated that in the absence of the central circadian clock of the SCN, peripheral tissues remain synchronized with the external light and dark cycle, indicating that without the control of the SCN, peripheral organs and tissues can still generate circadian rhythms to maintain physiological function. Studies using primary hepatocyte culture demonstrated that the tenuous connection between hepatocytes boosted enhances the local synchronization of peripheral tissue, stabilizing the circadian rhythm like the liver (Guenthner et al., 2014). However, over time, the rhythmic phases of peripheral organs and tissues fall out of sync (Yoo et al., 2004).

The circadian clock in the peripheral tissue is affected by external environmental factors that alter its cycle and phase. Factors that affect the function of the circadian clock are termed *zeitgebers*, and include the light-dark cycle, temperature, feeding, and social interaction (Ruiter et al., 2003; Roedel et al., 2006). The light-dark cycle is a major *zeitgeber* received by the SCN through a light signal input from an intrinsically photosensitive retinal neuron that expresses melanopsin (Berson, 2003). As such, light is of great importance in synchronizing the circadian clock in mammals and regulating their physiological functions.

### 2.2 Self-regulating feedback loops of the circadian clock

**FIGURE 1:**
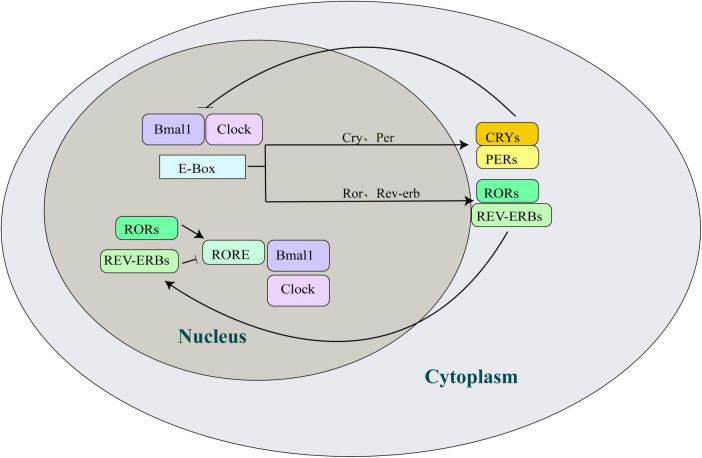
Overview of the circadian clock machinery. Bmal1 and Clock bind to the E-box elements and induce transcription of the Cry and Per genes. Subsequently, the complex formed by CRYs/PERs translocates into the nucleus and negatively feedback inhibits the transcriptional potential of Bmal1 and Clock, thereby inhibiting their own transcription. The activation of the orphan nuclear receptor genes, Ror and Rev-erb, by Bmal1 and Clock occurs in an interlocking cycle. Rev-erb and ROR competitively bind to the RRE in the Bmal1 promoter to inhibit and activate the expression of the Bmal1 gene, respectively. Bmal1, brain and muscle ARNT-like 1; Clock, circadian locomotor output cycles kaput; Cry, cryptochrome; Per, period; ROR, retinoic acid receptor related orphan receptor; Rev-erb, reverse erythroblastosis virus.

Circadian rhythms in mammals are governed by the transcription-translation feedback loops (TTFLs) of circadian clock genes, with a periodicity of approximately 24 h (Buijs and Kalsbeek, 2001; Albrecht and Eichele, 2003; Fagiani et al., 2022). The circadian clock system includes numerous genes and their transcribed and translated protein products that regulate the mammalian circadian clock, such as Bmal1 (brain and muscle ARNT-like 1), CLOCK (circadian locomotor output cycles kaput), Per (period, per1/per2), Cry (cryptochrome, cry1/cry2), nuclear receptor ROR (retinoic acid receptor related orphan receptor), and Rev-erb (Rev-erbα/Rev-erbβ), among others (Figure 1). Circadian rhythms are maintained by positive regulatory elements comprising CLOCK-Bmal1 heterodimers and two negative regulatory elements comprising the Per/Cry families. The CLOCK-Bmal1 heterodimer binds to E-box elements in the promoter and enhancer regions of both genes to positively regulate gene transcription, induce the transcription and translation of Per and Cry, and form protein products in the nucleus. The accumulated Per/Cry complex interferes with the transcription of CLOCK and Bmal1, inhibiting their transcription (Buijs and Kalsbeek, 2001). The reduction in Pers and Crys transcript levels triggers negative feedback inhibition, prompting CLOCK and Bmal1 to initiate a new gene expression cycle. In addition, the CLOCK-Bmal1 heterodimer activates the Rev-erb and ROR E-box, with ROR binding to the ROR/Rev-erb response element (RRE) in the Bmal1 promoter, regulating its inhibition and activation (Ueda et al., 2005). Periodic circadian oscillations result from the coordination of positive and negative regulatory elements.

## 3 The circadian clock in the cochlea

### 3.1 Structural functions of the cochlea

The cochlea, the auditory receptor in the inner ear of mammals, consists of four functional regions: the sensory epithelium, neuronal compartment, lateral wall, and immune cells (Milon et al., 2021). The sensory epithelium, an organ of Corti, contains both supporting cells (SCs) and hair cells (HCs). SCs are responsible for ion circulation and provide structural support and repair to the cochlea. HCs, including inner hair cells (IHCs) and outer hair cells (OHCs), where IHCs are responsible for sensing sound vibrations and converting sound vibrations into nerve impulses, and OHCs play the role of amplifying sound signals (Medina-Garin et al., 2016). Sensitivity to sound is closely linked to the condition of the sensory hair cells, which are vulnerable to damage or loss from a range of physical and chemical factors, such as noise or ototoxic drugs, resulting in a reduction or complete loss of hearing function.

### 3.2 Expression of circadian clock-related genes in the cochlea

Circadian clock-related genes are present in the SCN and peripheral tissues, though their expression levels may vary across tissues. A self-sustaining circadian rhythm system has been demonstrated in the mouse cochlea and inferior colliculus (IC) (Park et al., 2016). Meltser et al. (2014) discovered several mRNA transcripts of circadian clock-related genes in the mouse cochlea that regulate the generation and maintenance of circadian rhythm, including Per1, Per2, Bmal1, CLOCK and Rev-erb α. They found an inverse correlation between positive (Bmal1, CLOCK) and negative (Per1, Per2) regulatory elements. The transgenic PERIOD2:LUCIFEREASE (PER2:LUC) mouse strain allows the real-time monitoring of gene expression rhythms (Yoo et al., 2004). Using this model, Meltser et al. (2014) discovered that the cochlea, with abundant expression of circadian clock genes and the presence of circadian oscillatory rhythms, is capable of generating stable and self-sustained oscillations of PER2. In the cochlea, PER2 is predominantly expressed in the sensory hair cells of the organ of Corti and spiral ganglion neurons (SGNs), indicating that PER2 oscillations may originate from these cells (Park et al., 2017). Additionally, the rhythmic amplitude of the cochlear circadian clock is higher than that of the liver circadian clock (Meltser et al., 2014).

### 3.3 Effects of the circadian clock on cochlear hearing function

Circadian clock dysregulation can have deleterious effects on the auditory system. Light, a major *zeitgeber* of circadian rhythms, affects various physiological functions in mammals. In a study using light conditions to regulate circadian oscillations in the cochlea, Yang et al. (2020) found that circadian expression of the clock genes Per1, Per2, Bmal1, CLOCK and Rev-erb α was suppressed in the cochlea of CBA/CaJ mice exposed to constant light. However, while the circadian expression of Bmal1 and CLOCK was significantly lost, Per1, Per2 and Rev-erb α was still showed circadian expression in the cochlea of mice exposed to constant dark. This indicated that the circadian rhythm of the cochlea was more perturbed by constant light (Yang et al., 2020). This study also showed that constant light exacerbated the permanent threshold shift (PTS) induced by high-intensity, high-frequency noise and the loss of OHCs in the cochlea, indicating that dysregulation of the circadian clock in the cochlea may increase cochlear susceptibility to noise (Yang et al., 2020). Therefore, the combined effect of constant light and high-intensity, high-frequency noise exposure leads to circadian clock dysregulation and exacerbates NIHL (Yang et al., 2020). In addition, the severity of antibiotic-induced ototoxicity in rats has been linked to circadian sensitivity (Blunston et al., 2015). These studies suggest that the auditory system function is regulated by the circadian clock.

## 4 Circadian clock and effects of noise exposure on the auditory system

### 4.1 Molecular mechanisms of NIHL

The World Health Organization (2021) reports that nearly 2.5 billion people may experience hearing loss by 2050, with at least 700 million requiring rehabilitation. NIHL is a common forms of sensorineural hearing loss with complex underlying mechanisms. Currently, there is no effective treatment for complete reversal of NIHL (Nelson et al., 2005). Reducing cochlear damage requires investigating the mechanisms of NIHL and developing preventive and pharmacological measures to address its root causes.

The primary molecular mechanisms underlying NIHL include oxidative stress, inflammation, and apoptosis (Kurabi et al., 2017). Oxidative stress is a major contributor to the development of NIHL. Under high-intensity noise stimulation, Ca^2+^ overload occurs in the hair cells of the cochlea, NADPH oxidase is activated, and the mitochondria are induced to release reactive oxygen species (ROS) (Kurabi et al., 2017). Excessive ROS damages cochlear hair cells by reacting with intracellular DNA, proteins, cell surface receptors, and membrane lipids (Kamio et al., 2012). In addition, ROS can also activate the TLR-4/NF-κB and MAPK-c-JNK pathways, thereby inducing the production of inflammatory factors and apoptosis (Wang et al., 2007; Tan et al., 2016). Noise induces cochlear inflammation by activating damage-associated molecular patterns (DAMPs), and molecules released from damaged cells bind to DAMP receptors, inducing early pro-inflammatory cytokine (TNF-α and IL-1β) and chemokine (CCL2) production and late macrophage recruitment (Frye et al., 2019). Circulating GCs have been implicated in the modulation of the inflammatory responses in the cochlea (Cederroth et al., 2019). The secretion of GCs is regulated by the circadian clock and the central nervous system, and it has been reported that the removal of circulating GCs by adrenalectomy (ADX) eliminates the transcription of clock-controlled genes (CCGs) involved in inflammation, indicating that circulating GCs may play a role in cochlear inflammation by regulating the expression of inflammation-related genes (Cederroth et al., 2019).

### 4.2 The regulatory role of the circadian clock in NIHL

The sensitivity of the auditory system to noise trauma is regulated by factors, such as genetic factors (Lavinsky et al., 2015), stress (Canlon et al., 2007), and age (Basinou et al., 2017). Circadian rhythms can influence the pathophysiology of the auditory system, with research showing that auditory sensitivity to noise trauma varies depending on the time of day (Meltser et al., 2010; Meltser et al., 2014).

Meltser et al. (2010) showed that when exposed to noise of the same intensity and frequency, mice in the daytime noise exposure group developed temporary threshold shift (TTS), whereas mice in the nighttime noise exposure group developed PTS, indicating greater sensitivity to noise-induced damage to the auditory system during nocturnal activities. In addition, mice showed similar behavioral movements during the daytime and nighttime when they were exposed to noise, which could indicate that the circadian sensitivity of mice to noise occurs independent of their own behavior (Park et al., 2016). It has further shown that the recovery of ABR thresholds in mice depended on the time of noise exposure, while excessive noise exposure altered the circadian oscillation of mRNA transcripts of clock genes Per1, Per2, Rev-erb α, and Bmal1 genes in the cochlea of CBA/CaJ mice (Meltser et al., 2014). In addition, the amplitude of Per1, Per2 and Rev-erb α gene rhythms was suppressed, while the amplitude of Bmal1 gene rhythm was increased in the cochlea of mice receiving nocturnal noise exposure. One possible mechanism for the diurnal difference in noise trauma response is the role of cochlear neurotrophic signals. Neurotrophic factors are important regulators of cochlear synaptogenesis and plasticity.

## 5 Circadian regulations of the effects of noise in the auditory system by the Trk B-BDNF signaling pathway

### 5.1 Effects of noise on the BDNF expression and function

Noise exposure causes dendritic synaptic swelling in auditory neurons, resulting in temporary damage to the auditory system. Neurotrophin-3 (NT-3) and brain-derived neurotrophic factor (BDNF) are important neurotrophic factors involved in cochlear synaptogenesis (Ramekers et al., 2012), and playing important roles in cochlear development and adult auditory physiology. NT-3 is a crucial factor that regulates of neuronal survival in the auditory system and further plays a role in cochlear hearing loss (Yang et al., 2011). In the cochlea of neonatal mice, BDNF is primarily expressed in vestibular hair cells and has a protective effect on auditory neurons, and attenuates noise-induced hearing loss by promoting neuronal growth and repair, as well as facilitating the proliferation and differentiation of a wide range of cells in the inner ear (Yang et al., 2011; Singer et al., 2014). Zuccotti et al. (2012) used conditional knockout mice to show that BDNF is essential for maintaining adult IHC transmitter release sites and opposing afferents in high-frequency turns. They found that lacking BDNF hampers IHC synapse physiology, but protects against NIHL.

**FIGURE 2:**
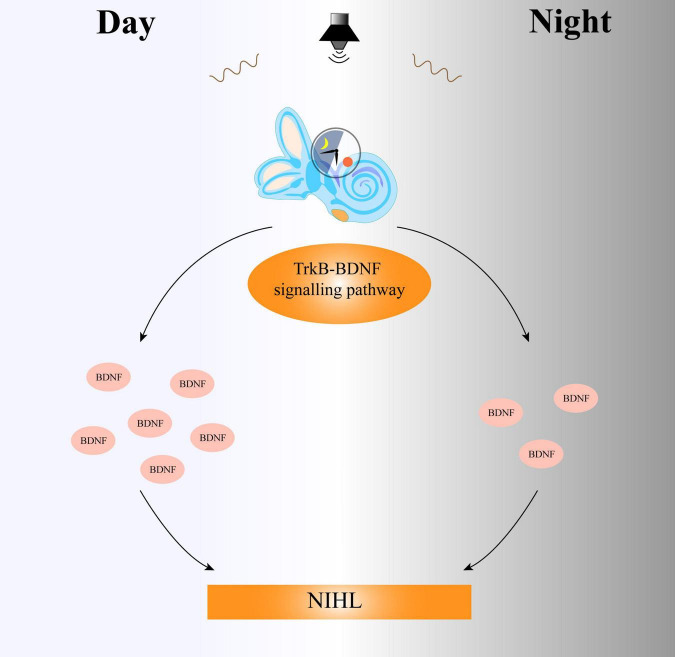
The circadian clock system regulates the sensitivity of the cochlea to day and night through the TrkB-BDNF signaling pathway. Daytime noise exposure leads to an increase in the expression of BDNF, while the secretion of BDNF in the cochlea decreases after nighttime noise exposure. Thus, the difference in BDNF expression between day and night results in different degrees of auditory damage. TrkB, tropomyosin related kinase type B; BDNF, brain-derived neurotrophic factor; NIHL, noise-induced hearing loss.

BDNF stimulates regeneration in various systems and is released following injury (Fernandez et al., 2021). BDNF transcriptional induction differs after exposure to the same noise during the daytime and nighttime. The high secretion of BDNF during the daytime has a strong ability to repair noise-induced cochlear damage, while the transcription level of BDNF is low and the secretion is low at night, and the cochlea cannot trigger a protective response dependent on BDNF and affect the auditory function (Meltser et al., 2014). The lack of BDNF induction after nocturnal noise exposure may further increase the sensitivity of the cochlea to noise, indicating that the protective role of BDNF in the cochlea may be indispensable for maintaining normal auditory function (Figure 2).

### 5.2 Effect of circadian rhythm on BDNF expression

Circadian rhythm regulates gene expression in the central system through the Bmal1-CLOCK positive feedback loop (Ishii et al., 2017). BDNF is also abundantly expressed in tissues such as the prefrontal cortex, hippocampus, and liver (Bilu et al., 2022). Under normal light-dark cycling conditions, the expression of BDNF in the hippocampal region of mice during the daytime was significantly higher than that during the nighttime, indicating that the circadian rhythm may affect brain function by regulating the expression of BDNF (Genzer et al., 2016). In addition, AMPK and mTOR regulate circadian rhythms and are involved in BDNF signaling. The circadian rhythm may influence BDNF expression by modulating the AMPK-mTOR pathway, with activation or inhibition promoting BDNF synthesis and release (Genzer et al., 2016).

### 5.3 The circadian rhythm mediates the effects of noise in the auditory system via the TrkB-BDNF signaling pathway

Tropomyosin related kinase type B (TrkB) is involved in neuronal development and plasticity, and BDNF regulates circadian clock rhythmicity by affecting the expression and transcription of circadian clock genes through its action on TrkB (Ishii et al., 2019). TrkB acts as a receptor for BDNF and can activate downstream signaling pathway to further exert the biological effects of BDNF. Differences in transcriptional expression of BDNF-mediated signals throughout the day suggest that the circadian system regulates the sensitivity of the cochlea to daytime and nighttime noises through the TrkB-BDNF signaling pathway (Figure 2). Mature BDNF preferentially binds to TrkB, and its activation prevents NIHL. The selective agonist of TrkB, 7, 8-dihydroxyflavone (DHF), plays an important protective role in the process. Meltser et al. (2014) also found that exposure of cochlear explants to DHF resulted in increased amplitude, phase delay, and changes in the PER2:LUC activity cycle. The hearing of mice exposed to noise trauma at night was also restored following DHF injection.

The TrkB agonist drug amitriptyline (AT) significantly impacts the regeneration of afferent cochlear synapses following noise-induced synaptopathy. Fernandez et al. (2021) showed that *in vitro* co-culture experiments with SGNs and organ of Corti, cochlear afferent synapses were regenerated in cultures, and their number was significantly increased by treatment with 0.5 μmol/L AT for 6 days, and in *in vivo* experiments, AT also improved synaptic and auditory function. It has been reported that when CBA/CaJ mice were exposed to octave band noise (8–16 kHz, 100 dB SPL) for 2 h, reversible hearing threshold displacement and permanent synaptic damage occurred, although this did not result in an acute loss of hair cells (Fernandez et al., 2021). Topical AT after noise exposure restored cochlear nerve function and inner hair cell synapses, and systemic administration before noise exposure preserved cochlear synapses and auditory function *in vivo*; this protective effect of AT was still present after 1 year of treatment (Fernandez et al., 2021). AT significantly affects the regeneration of intracochlear inter hair cell synapses. AT and other related drugs are important in the clinical treatment of hereditary hearing loss.

## 6 Circadian rhythms of the HPA axis modulate the effects of noise exposure on the auditory system

### 6.1 Interaction of the HPA axis and circadian rhythm

The hypothalamic-pituitary-adrenal (HPA) axis is a key component of the neuroendocrine system, regulating the body’s stress response and various physiological activities, including circadian rhythms, metabolism, and inflammatory (Russell and Lightman, 2019). The secretion of GCs is regulated by the central circadian clock of the SCN and naturally fluctuates in the circadian rhythm, with the highest levels at the beginning of the active phase of diurnal animals and the lowest levels during the inactive phase (Ishida et al., 2005; Shimba and Ikuta, 2020). GCs exert their effects through specific nuclear receptors, including glucocorticoid receptors (GR) and mineralocorticoid receptors (MR) (Kil and Kalinec, 2013). The HPA axis activity is controlled by the circadian clock, which regulates GCs levels within a physiological range suitable for system homeostasis by utilizing feedforward and feedback loops (Focke and Iremonger, 2020). Adrenal corticotropin (CORT) secretion, a precursor of GCs, is controlled by corticotropin-releasing hormone (CRH) neurons located in the paraventricular nucleus (PVN) of the hypothalamus (Focke and Iremonger, 2020). GR and MR expression have been observed in various cochlear cells (Kil and Kalinec, 2013). Circadian rhythm exists at all levels of the HPA axis, and appears to cooperate with endogenous adrenal circadian rhythms to mediate diurnal variations in CORT secretion over a 24-h period.

The circadian clock system is affected by the HPA axis in organs and tissues (Balsalobre et al., 2000). However, the central circadian clock, which maintains an intrinsic circadian rhythm, is less affected by GCs, as the SCN does not express GR (Balsalobre et al., 2000). In response to stressors, GCs mediate the cyclic resetting of the peripheral circadian clock, adapting to environmental changes by regulating the expression of clock-related genes in the peripheral tissues, thus maintaining a steady state in the organism (Burioka et al., 2007). It has further been found that the tandem glucocorticoid response element (GRE) promoter of the Per1 gene induced GCs in peripheral tissues, such as the liver and kidney, to mediate Per1 mRNA expression and stimulated Per2 mRNA expression in a GRE-dependent manner (Yamamoto et al., 2005; Segall et al., 2009). Additionally, CLOCK-Bmal1 is an inverse regulator of GC activity in peripheral tissues and organs, directly regulating GR transcriptional activity through acetylation and antagonizing circulating GCs (Nader et al., 2009; Nader et al., 2010).

### 6.2 Changes in circadian rhythm in cochlear GCs-dependent signaling pathway

**FIGURE 3:**
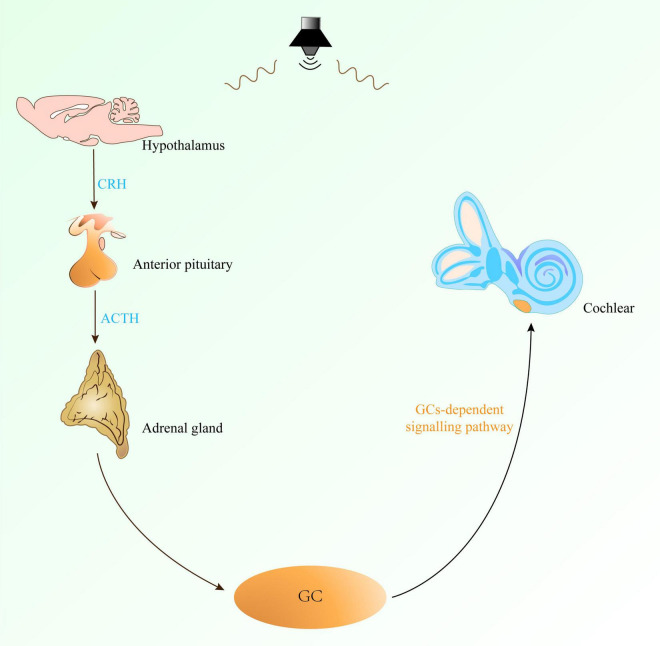
The GCs-dependent signaling pathway regulates the sensitivity of the cochlea to noise. The GCs-dependent signaling pathway involves the HPA axis. Noise exposure can prompt the hypothalamus to secrete CRH, which acts on the pituitary gland to stimulate it to secrete ACTH, and then stimulates the adrenal cortex to secrete GC. GC reaches the cochlea through the blood circulation, thereby changing the sensitivity of the cochlea to noise and affecting the occurrence and development of NIHL. CRH, corticotropin-releasing hormone; ACTH, adrenocorticotropic hormone; GC, glucocorticoids; HPA axis, hypothalamus-pituitary-adrenal gland axis.

Complex tissues and cells in the cochlea form the basis of its sensory function. Sensory hair cells cannot regenerate while supporting cells maintain local homeostasis. These cells are sensitive to perturbations, involved in inflammatory signaling, and influence auditory function through various physiological effects. Acoustic trauma has been demonstrated to activate the HPA axis, resulting in increased plasma release of ACTH and CORT (Tahera et al., 2007; Lee et al., 2019). CORT, an end product of the HPA axis, is the major GC reflecting the stress response, and GCs in the blood are transported with the circulatory system to various parts of the body, including the cochlea (Figure 3). In rodents, corticosterone is the primary bioactive corticosteroid. Its levels and circadian cycling in the cochlea positively correlate with susceptibility to NIHL and pro-inflammatory signaling (Barnes et al., 2023).

Cochlear sensitivity to noise is regulated by GCs-dependent signaling pathway (Figure 3). GR is detected in hair cells, supporting cells, spiral ligaments, and the stria vascularis, suggesting its role in regulating sensory and non-sensory cochlear tissues (Sarlus et al., 2019). Cederroth et al. (2019) found that GCs regulate auditory function, and that GCs signaling can be influenced by various stimuli, like stressors and drugs, which alter the circadian pattern of GC secretion. Removal of circulating GCs of cochlea by ADX did not significantly affect the expression of core clock genes but altered the transcript levels of CCGs involved in inflammation, indicating that GCs regulate the circadian expression of inflammation-related genes in the cochlea. The diurnal expression of inflammation-related genes in the cochlea protects auditory function, while the increased of pro-inflammatory signals at night suggest that GCs-regulated genes are activated only at night, making the cochlea most sensitive to noise (Cederroth et al., 2019).

Removal of circulating GCs by ADX affected inflammation-related CCGs, although GCs had no significant effect on the expression of core circadian genes, suggesting that GCs regulate the circadian expression of inflammation-related genes and protect auditory function. The increase in pro-inflammatory signals in the cochlea at night indicates that a subset of genes regulated by GCs are activated only during this time, making the mouse cochlea most sensitive to noise at night (Cederroth et al., 2019). However, the synthetic glucocorticoid dexamethasone (DEX), administered during the daytime when circulating glucocorticoids are low, effectively prevents injury from acute noise trauma (Cederroth et al., 2019). A comprehensive understanding of the circadian regulation of noise sensitivity in GCs offers novel insights into the field of studying temporal pharmacology-based treatments for NIHL.

### 6.3 The GCs-dependent signaling pathway regulates the sensitivity of the cochlea to noise

Barnes et al. (2023) showed that MR ablation of SCs altered the cochlea’s physiological response to noise, reducing the instantaneous ABR P1 amplitude and causing synaptic ribbon loss following mild noise exposure without eliciting noise-induced immune responses. GCs modulate auditory sensitivity, and while support cell GR ablation improves ABR threshold sensitivity, it impairs the recovery of the ABR threshold following mild noise exposure. These findings illustrate the differences in the roles of MR and GR expression in cochlear supporting cells in the basal, resting, and recovery states after noise exposure (Barnes et al., 2023).

It is well known that the inner ear is not an immune-privileged organ, while the blood-labyrinthine barrier allows low concentrations of antibodies and lymphocytes to pass through, triggering an immune response in the inner ear. The cochlea primarily induces an inflammatory response to noise trauma. Studies have shown that GCs downregulate inflammatory responses through multiple mechanisms (Ronchetti et al., 2018). By binding to positive GRE, it directly upregulates anti-inflammatory cytokines (such as IL-10) levels and down-regulate the levels of pro-inflammatory cytokines (such as IL-1, IL-6, and TNF-α), which are important for inflammatory regulation (Ronchetti et al., 2018). Changes in the circadian clock system can affect glucocorticoid homeostasis, and the interaction between Cry and GR is enhanced by GC agonists, like DEX (Lamia et al., 2011). The addition of DEX prevents the attenuation of rhythmic PER2:LUC expression over time, and the process of GR-mediated transcription induced by DEX can be blocked by Cry1, demonstrating that Crys plays a direct role in regulating GR function, thereby regulating the GCs production (Sarlus et al., 2019). DEX also exerted a protective effect against noise trauma within 24 h after noise exposure at lower GC levels, while administration at higher GC levels did not provide protection. This may be due to differences in local bioavailability in the cochlea, influenced by variations in the permeability of the blood-labyrinth barrier (Cederroth et al., 2019).

## 7 Conclusion

Clinical treatments for hearing loss generally involve hearing aids and cochlear implants. However, this alternative treatment does not fundamentally repair damaged hair cells and restore full function to the spiral ganglion. The evidence collected in this review indicates that the circadian clock system within the auditory system may regulate noise sensitivity throughout the day. The rhythmic expression of circadian clock genes results in differences in cochlear sensitivity to noise, which in turn affects the development of noise induced hearing loss. Its mechanism, involving the TrkB-BDNF signaling pathway and GCs, offers novel ideas for the targeted treatment of NIHL. Furthermore, it remains to be determined whether altered cochlear circadian rhythms increase susceptibility to broad-spectrum auditory insults other than noise damage, such as ototoxic drugs (Tserga et al., 2020). Currently, there has been a paucity of research on chronopharmacology for the treatment of NIHL. Consequently, further investigation is required into the relationship between the cochlear circadian clock system and NIHL, and the mechanisms by which medication administered at different times of day affects NIHL. It may help to provide new ideas for the prevention and treatment of NIHL.
